# Complete genome sequence of *Pseudomonas aeruginosa* phage Knedl

**DOI:** 10.1128/mra.01174-23

**Published:** 2024-02-23

**Authors:** Enea Maffei, Christina Manner, Urs Jenal, Alexander Harms

**Affiliations:** 1 Biozentrum, University of Basel, Basel, Switzerland; 2 Institute of Food, Nutrition and Health, ETH Zurich, Zurich, Switzerland; Queens College Department of Biology, USA

**Keywords:** bacteriophages, biofilms, receptors, bacteriophage genetics

## Abstract

Bacteriophage Knedl is the first reported *Pseudomonas aeruginosa* phage that targets the Psl exopolysaccharide as receptor. Here, we report the genome of Knedl, demonstrating that it belongs to the genus *Iggyvirus* of the *Queuovirinae* subfamily. Future studies on the infection mechanism of Knedl could inform phage-based approaches to eradicate biofilms.

## ANNOUNCEMENT

**Fig 1:**
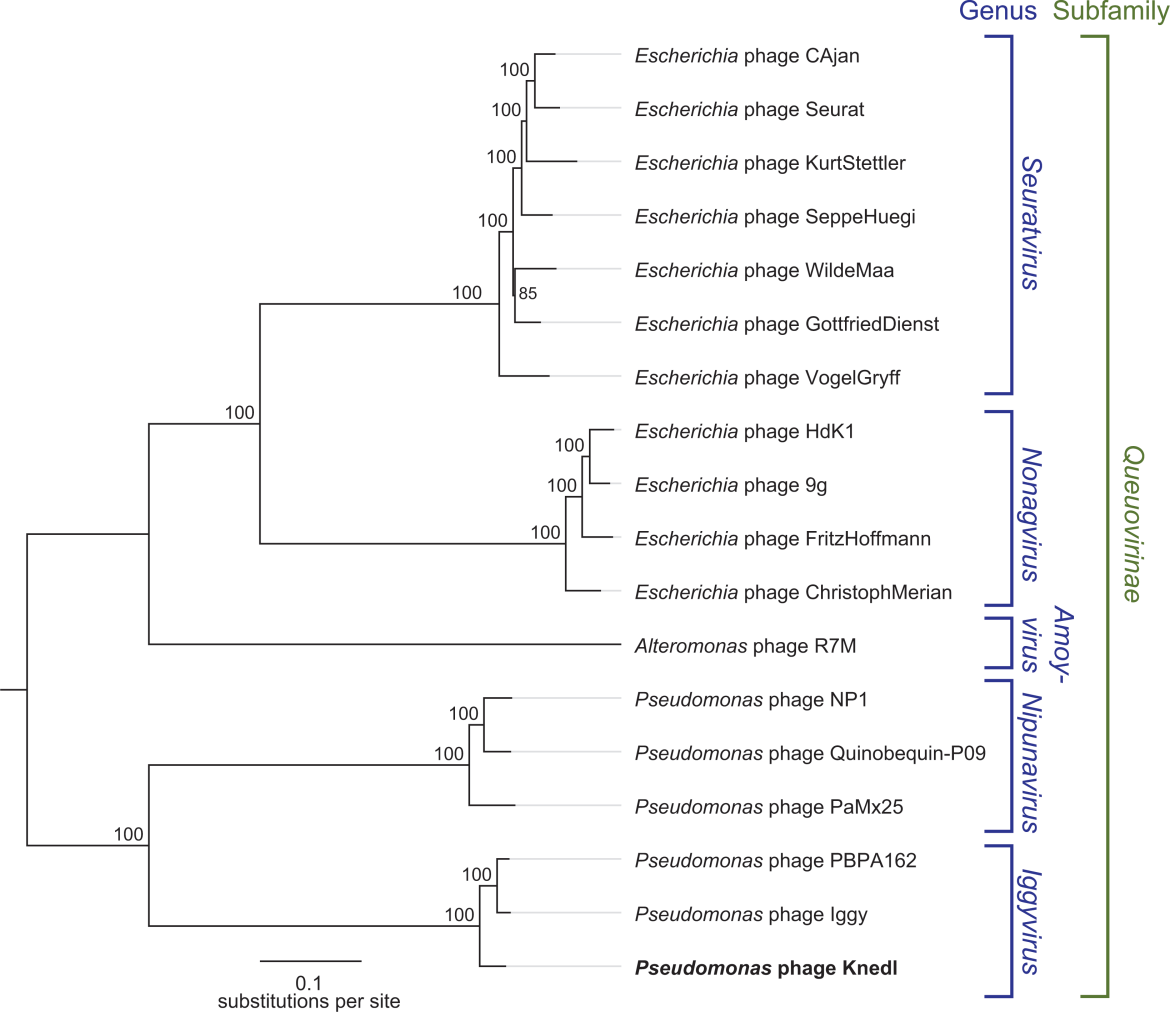
Whole genome phylogeny of phage Knedl. Genomes of phages from relevant genera and infecting various hosts of the *Queuovirinae* were retrieved from NCBI GenBank (release 258) and aligned using MAFFT (v1.5.0, alignment available at 10.6084/m9.figshare.25062299). Following manual curation in Geneious Prime v 2023.1.1, the maximum likelihood phylogeny of Knedl was calculated using PHYML (v2.2.4) implemented in Geneious Prime. The phylogeny was rooted between the *Pseudomonas*- and *Escherichia*-infecting phage genera. *Pseudomonas* phage Knedl is highlighted in bold.

Most bacteriophages infecting *Pseudomonas aeruginosa* target type IV pili or lipopolysaccharides as receptors to initiate infection, with the notable exception of phage OMKO1 which uses the outer membrane protein OprM as host receptor (1–3). A recent report suggested that phage Knedl, a small siphovirus infecting *P. aeruginosa* laboratory strain PAO1, exploits the extracellular polysaccharide Psl as receptor to initiate infection (4, 5). We now expand on this finding by reporting the complete genome sequence of this phage. Phage Knedl was isolated in spring 2021 from sewage water from the city of Basel by plating on a host grown at 37°C shaking carrying a plasmid for the overexpression of *hecE* (PA2781). Production of cyclic diguanylate monophosphate (cyclic di-GMP) (due to overexpression of *hecE*) was induced by addition of Isopropyl β-D-1-thiogalactopyranoside (IPTG) to the top agar. Following the initial isolation, candidate phages were streaked on the wild-type host (i.e., with regular cyclic di-GMP level) and the isolation strain overexpressing *hecE* to assess the dependence of the phage on cyclic di-GMP levels (4). Plaque size and turbidity on the two hosts were compared, and phages showing larger plaques on the isolation strain were further characterized, ultimately leading to the identification of phage Knedl (4). Subsequently, phage Knedl was passaged three times to ensure stock purity, and a high-titer lysate was generated by double-agar and Sodium Magnesium buffer (SM) buffer overlays, similar to the method described previously (5). Comparing phage Knedl’s infectivity on various extracellular polysaccharide mutants led to the identification of Psl as the receptor (4). We then extracted the genomic DNA of Knedl from the lysate using the extended protocol of Norgen Biotek Phage DNA isolation kit (5, 6). The sequencing library was prepared using an Illumina Nextera kit and sequenced as paired-end reads with the Illumina NextSeq 550 platform. Trimmed sequencing reads (151-bp length) were assembled using the Geneious Assembler implemented in Geneious Prime 2021.0.1, resulting in a circular assembly of 59,205 bp with a GC content of 56.2% and a mean coverage of 71×. We annotated the Knedl genome using Pharokka v.1.3.2. as implemented in Galaxy Europe (usegalaxy.eu) with standard settings (7). The Pharokka annotation suggests that Knedl codes for 90 proteins and no transfer RNA (tRNAs) (7). A whole genome BLAST (v. 2.12.0) against the NCBI nucleotide database (release 258) revealed that Knedl belongs to the proposed genus of *Iggyvirus* of the *Queuovirinae* subfamily, with the top hit being *Pseudomonas* phage Iggy (92.36% nucleotide identity across the whole genomes) (8, 9). We then retrieved representative sequences covering relevant genera and hosts of the *Queuovirinae* from NCBI GenBank (release 258) to construct a whole genome phylogeny (Fig. 1) and confirmed that phage Knedl is a member of the *Iggyvirus* genus. Closer inspection of the genome revealed that (like other *Queuovirinae*) phage Knedl possesses the genes required to modify its genomic 2′-deoxyguanines into 2-deoxy-7-cyano-7-deazaguanine (dPreQ_0_), similar to phage Iggy (9, 10).

Taken together, phage Knedl is the first *P. aeruginosa* phage known to specifically target biofilm matrix components as host receptor, and further studies on the precise infection mechanism may provide valuable insight into how phages could be used to eradicate biofilms in the context of bacterial infections.

## Data Availability

This whole genome shotgun project has been deposited under the BioProject PRJNA1040154. The genome of Knedl has been deposited on GenBank under the accession number OR805297.1. The version described in this paper is the first version. The whole sequencing reads have been deposited on SRA under the accession number SRX22580910.
